# Criticism

**Published:** 1880-07

**Authors:** 


CRITICISM.—Not knowing "L." of the Missouri Dental Journal, we presume that he is "L(ord) of all he surveys," minus the titles D. D. S. and M. D. We are willing to award to him the degree of "A. S. S." to which he alludes.

We are sorry that *he* is unable to *see*, "what use?" We are happy to inform him that gentlemen of acknowledged ability have expressed the great need of such a reference work as we propose to make of "The Dental Directory of the World," which lie beyond the perception of his critical optics. From "L" (ord's) remarks, one would conclude that he was without the five dollars which would entitle him to a place among the *heavy letter* gentlemen. If such is the case we will most cheerfully print his name in that type, gratis; and also give him credit for his "snap" in the Missouri Dental Journal, with whatever titles he may furnish us; but let us turn from "this picture" to what is said by Prof. J. Taft, who is *high authority* in dental matters. This distinguished gentlemen copies our communication into the *Dental Register*, o which he is editor, and follows it with the succeeding remarks, viz.: "The enterprise indicated by the above communication is one of importance and interest to every dentist of this country, and to those of other countries as well.

"This as it seems, is not to be a complete history of dentistry but a full reference work in respect to persons and things.

"This is a work that cannot be accomplished without the hearty co-operation on the part of the profession, no man un-aided can do this work as it should be done, so then let every one give his modicum of help. Such a work complete and accurate would find a possessor in every dentist in the world — who speaks the English language at least — who has any professional aspirations at all.

"Such a work as this, followed by a complete history of the profession, would constitute an invaluable addition to the special literature of dentistry, and it is to be hoped that ere long this want will be supplied; it will if the profession gives the proper aid and co-operation; the which let every one do according to his ability."

'Tis well to have a title or degree from a reputable Medical or Dental College or institution, but while this is the case, there are many estimable members of the profession who have never received either. We hope therefore, that none will be deterred from sending in their names, &c., &c., as requested. Please read the communication in this journal, page 166, (condensed on 4th cover page,) and give the desired information *now*. B. M. W.

